# Supplementary material for: Bayesian refinement of protein structures and ensembles against SAXS data using molecular dynamics
Source: PLoS Comput Biol. 2017 Oct 18;13(10):e1005800. doi: 10.1371/journal.pcbi.1005800 (PMC5662244; doi:10.1371/journal.pcbi.1005800)
Supplement: S1 Archive — (BZ2) [file pcbi.1005800.s011.tar.bz2 › gromacs_4.62_bayesian_saxs_refi/share/html/online/protunf.html]

Getting started - Protein unfolding


| Getting started - Protein unfolding Main Table of Contents |

**VERSION 4.6  
Sat 19 Jan 2013**

---

### Protein unfolding

In this exercise we will study a protein unfolding simulation that was
done before. The protein is the C-terminal fragment of the L7/L12
ribosomal protein (see below). It consists of 68 residues, and is
known to be quite stable (in simulations). It is dissolved in a box
filled with 3777 water molecules a structural Sulfate ion and four
Sodium ions. A simulation was performed for 10 ns at 400 K. The
trajectory and other relevant files can be found in
~david/ctf.

|  |  |
| --- | --- |
|  |  |
| Native structure | 200 ps |
|  |  |
| 500 ps | 1 ns |
|  |  |
| 4 ns | 10 ns |

  

---

  

### Analysis

1. Start by making a new working directory, and then move there.
     
     

   |  |  |
   | --- | --- |
   |  | cd ~/tutor |
   |  | mkdir unfold |
   |  | cd unfold |
2. View the trajectory on your own X-screen (program
   ngmx).
     
     

   |  |  |  |
   | --- | --- | --- |
   |  | ngmx -s ~david/ctf/unfold.tpr -f ~david/ctf/unfold.xtc   \| |

     
   *Hint 1: In the filter it may be advantageous to select Mainchain
   rather than Protein.  
   Hint 2: Go to the display menu and select options. Then set skip frames
   to 9 before you start the animation.*
   What happens to the protein?
3. The Root Mean Square Deviation (RMSD) with respect to the crystal
   structure (program
   g\_rms) is a measure of how well the
   crystal (starting) structure is maintained in the simulation.
     
     

   |  |  |  |
   | --- | --- | --- |
   |  | g\_rms -s ~david/ctf/unfold -f ~david/ctf/unfold -o rmsd   \| |

     
   Select the 1 for the number of groups, and select C-alpha (group 3) for fitting
   and for computing the RMSD. View the output graph with xmgrace.
     
     

   |  |  |  |
   | --- | --- | --- |
   |  | xmgrace rmsd.xvg   \| |

     
   Does the RMSD
   converge within the simulation? If not, what does this indicate?

   - The Radius of Gyration (Rg, program
     g\_gyrate)) is a measure of the size of the
     protein.
       
       

     |  |  |  |
     | --- | --- | --- |
     |  | g\_gyrate -p -s ~david/ctf/unfold -f ~david/ctf/unfold -o gyrate   \| |

       
     Select protein when asked. View the graph with xmgrace:
       
       

     |  |  |  |
     | --- | --- | --- |
     |  | xmgrace -nxy gyrate.xvg   \| |

       
     Does the radius of gyration change during the
     simulation? The x, y, and z components indicate the
     overall shape of the molecule (like the axes of an ellipsoid).
     i.e. if they are all equal,
     the molecule has spherical shape, if one is much long than
     the other two, the molecule is elongated.
     Based on this graph and the animation
     does the protein change shape?

     - The Ramachandran Plot shows whether the backbone torsion angles
       (φ/ψ) of your
       peptide are within the allowed region.
       (program g\_rama).
       We will compare the start structure and the final structure by running
       the program twice.
         
         

       |  |  |  |
       | --- | --- | --- |
       |  | g\_rama -s ~david/ctf/unfold -f ~david/ctf/unfold -o rama-start -e 1   \| |
       |  | g\_rama -s ~david/ctf/unfold -f ~david/ctf/unfold -o rama-end -b 9999   \| |

         
       View the graphs with xmgrace:
         
         

       |  |  |  |
       | --- | --- | --- |
       |  | xmgrace rama-start.xvg rama-end.xvg -legend load   \| |

         
       In black we have the backbone angles from
       the starting structure, in red those from the final structure.
       *Hint 3: click on the red graph, and a dialog box will plop up.
       Select linetype none for the second graph, and select a circle as a symbol.*
       Are all the angles in the allowed region?
       What kind of structures do the angles indicate in the folded respectively unfolded conformation?

       - Now we will analyse the number of hydrogen bonds the protein makes.
         First with itself, then with the solvent.
           
           

         |  |  |  |
         | --- | --- | --- |
         |  | g\_hbond -s ~david/ctf/unfold -f ~david/ctf/unfold -num hbnum-pp   \| |

           
         Select protein as the first group and second group. Then redo the
         analysis for protein with solvent (change the output file name to
         hbnum-ps, and select first the protein, and then solvent).   
         View the output file:
           
           

         |  |  |  |
         | --- | --- | --- |
         |  | xmgrace hbnum-pp.xvg hbnum-ps.xvg   \| |

           
         Does the number of hydrogen bonds change for either of these?
       - Here we will analyse the solvent accessible surface area of the protein.
         We will be looking at both hydrophobic surface area and hydrophilic surface
         area.
           
           

         |  |  |  |
         | --- | --- | --- |
         |  | g\_sas -s ~david/ctf/unfold -f ~david/ctf/unfold -n ~david/ctf/index -skip 25   \| |

           
         (Select protein again). View the output file:
           
           

         |  |  |  |
         | --- | --- | --- |
         |  | xmgrace -nxy area.xvg   \| |

           
         How do the two components of the solvent accessible surface
         area change? How does the total change?
       - Secondary Structure analysis (program
         my\_dssp).
         This analysis uses the dssp (dictionary of secondary structure in proteins,
         Kabsch & Sander, 1983) software.
           
           

         |  |  |  |
         | --- | --- | --- |
         |  | my\_dssp -s ~david/ctf/unfold -f ~david/ctf/unfold -dt 50   \| |

           
         Select protein when asked to select a group.
         You can postprecess the output file with:
           
           

         |  |  |  |
         | --- | --- | --- |
         |  | xpm2ps -f ss.xpm -o ss.eps   \| |

           
         This will give you a postscript file which you can either print or
         view with xpsview.
           
           

         |  |  |  |
         | --- | --- | --- |
         |  | xpsview ss.eps   \| |

           
         What happens to the Alpha helix (in blue)? What happens to the Beta sheets? Which secondary structure element is more stable?

- Give a summary of what happens during the
  unfolding process. What happens first to the structure? How do the
  structure and shape of the protein develop? Try to formulate relevant
  conclusions for the protein folding problem based on this simulation.
  

---
